# A Novel Effector Protein of Apple Proliferation Phytoplasma Disrupts Cell Integrity of *Nicotiana* spp. Protoplasts

**DOI:** 10.3390/ijms20184613

**Published:** 2019-09-18

**Authors:** Cecilia Mittelberger, Hagen Stellmach, Bettina Hause, Christine Kerschbamer, Katja Schlink, Thomas Letschka, Katrin Janik

**Affiliations:** 1 Applied Genomics and Molecular Biology, Laimburg Research Centre, 39040 Auer/Ora (BZ), Italy; 2 Jasmonate Function & Mycorrhiza, Leibniz Institute of Plant Biochemistry, 06120 Halle, Germany

**Keywords:** phytoplasma, effector protein, apple, pathogenicity, virulence, apple proliferation

## Abstract

Effector proteins play an important role in the virulence of plant pathogens such as phytoplasma, which are the causative agents of hundreds of different plant diseases. The plant hosts comprise economically relevant crops such as apples (*Malus × domestica*), which can be infected by ‘*Candidatus* Phytoplasma mali’ (P. mali), a highly genetically dynamic plant pathogen. As the result of the genetic and functional analyses in this study, a new putative P. mali effector protein was revealed. The so-called “Protein in *Malus* Expressed 2” (PME2), which is expressed in apples during P. mali infection but not in the insect vector, shows regional genetic differences. In a heterologous expression assay using *Nicotiana benthamiana* and *Nicotiana occidentalis* mesophyll protoplasts, translocation of both PME2 variants in the cell nucleus was observed. Overexpression of the effector protein affected cell integrity in *Nicotiana* spp. protoplasts, indicating a potential role of this protein in pathogenic virulence. Interestingly, the two genetic variants of PME2 differ regarding their potential to manipulate cell integrity. However, the exact function of PME2 during disease manifestation and symptom development remains to be further elucidated. Aside from the first description of the function of a novel effector of P. mali, the results of this study underline the necessity for a more comprehensive description and understanding of the genetic diversity of P. mali as an indispensable basis for a functional understanding of apple proliferation disease.

## 1. Introduction

Phytoplasma are small, biotrophic bacteria that cause hundreds of different plant diseases and are involved in their infection cycle not only in plant hosts, but also in insect vectors. ’*Candidatus* Phytoplasma mali’ (P. mali), the causal agent of apple proliferation (AP) disease, has caused significant economic losses in apple production in Northern Italy (one of Europe’s main production areas) in the last decades [1]. Phytoplasma are obligate plant and insect symbionts that exhibit a biphasic life cycle comprising reproduction in certain phloem-feeding insects as well as in plants [2,3]. Within their plant host, phytoplasma colonize the phloem. By ingestion of phloem sap, insect vectors acquire the phytoplasma, with the colonization of those insects enabling the transmission of the pathogen between host plants [3,4]. Although several concepts of phytoplasma effector biology were able to be unraveled for the ’*Candidatus* Phytoplasma asteris’ strain Aster Yellow Witches’ Broom (AY-WB) in the model plant *Arabidopsis thaliana* [5,6,7,8,9,10,11,12], the understanding of effector-driven changes induced by P. mali remain limited. Genetic and functional homologues of AY-WB phytoplasma protein SAP11 could be identified in P. mali [10,13]. Recently a novel effector was described that exhibits E3 Ubiquitin ligase function and affects the plant’s basal defense [14]. Furthermore, the immunodominant membrane protein Imp of P. mali was shown not to be involved in symptom development but is considered to play a role during plant colonization [15]. A role of phytoplasmal HflB proteases and an AAA+ ATPase in AP virulence has been hypothesized but not yet clarified [16,17,18]. P. mali encodes genes for a Sec-dependent protein secretion system, whereas genes encoding components of other secretion systems, such as the type three secretion system, are mainly lacking [19,20]. Secreted phytoplasma proteins may directly interact with cellular host components and thus manipulate the cell’s metabolism [3]. Potential effector proteins may thus be identified by the presence of a characteristic *N*-terminal secretion signal.

The aim of this study was to characterize the function of the phytoplasmal “Protein in *Malus* Expressed 2” (PME2) from P. mali that exhibits genetic features indicating that it acts as an effector protein in plants. To unravel PME2s potential role as an effector, this study analyzed (1) whether it is genetically conserved; (2) whether it is expressed during infection; (3) where it is translocated within the plant cell; and (4) if it induces morphological changes within the expressing plant cells.

To address these questions, we analyzed the expression of PME2 in P. mali-infected *Malus × domestica* leaf and root tissue, and in infected *Cacopsylla picta* (i.e., insect species transmitting P. mali). In infected *Malus × domestica* we found two distinct genetic variants of *pme2*. In addition, heterologous overexpression of PME2 in mesophyll protoplasts of *Nicotiana* spp. was used to gain insights into the subcellular localization of PME2 as well as its effects on plant cell integrity. These data were complemented by the expression of PME2 in yeast. With the data presented here, the first steps into unraveling the molecular mechanism of PME2 function were taken, but further experiments in the future will be indispensable.

## 2. Results

### 2.1. In Silico Analysis of PME2 Indicates Effector Potential

**Figure 1:**
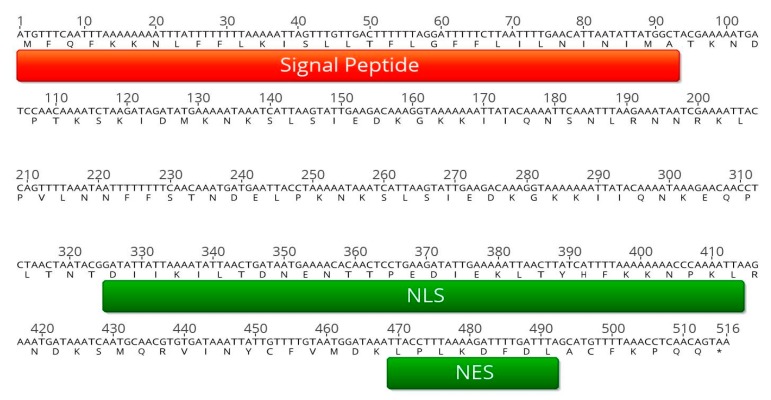
Results of the in silico analysis. Sequence analysis of *atp_00136* revealed the presence of an *N*-terminal signal peptide (indicated in red), as well as a nuclear localization signal (NLS), and a C-terminal nuclear export signal (NES), both indicated in green. Graphs were generated with Geneious Prime 2018 version 11.1.4.

Bioinformatic analysis of conserved hypothetical proteins encoded in the P. mali genome [19] revealed that CAP18323.1, encoded by the gene *atp_00136,* contains interesting features that might confer effector function. Neural networks and hidden Markov prediction models (Transmembrane Helices Hidden Markov Model; TMHMM) were applied to analyze CAP18323.1 for the presence of a signal peptide and the presence of transmembrane regions (SignalP v. 3.0 [21], TMHMM [22]). Since phytoplasma phylogenetically belong to Gram-positive bacteria [3], a prediction algorithm trained on this bacterial group was applied. The N-terminal amino acid-stretch 1–31 contains a signal peptide that is supposed to confer Sec-dependent secretion of the protein (Figure 1). Further transmembrane regions were not predicted, indicating that CAP18323.1 is not inserted in a membrane. Upon translation, *N*-terminal signal peptides are cleaved [23]. At the C-terminal part of CAP18323.1 an importin α/β-dependent nuclear localization site (NLS) and a nuclear export signal (NES) were predicted [24,25]. The absence of transmembrane regions in the mature protein, the predicted localization in the plant cytoplasma or the nucleus (WoLF PSORT, [26]), and the small size of about 16 kDa (Analysis Tool on the ExPASy Server, [27]) indicate that CAP18323.1 may exhibit an effector function (Figure 1).

### 2.2. *Atp_00136 (Pme2)* is Expressed in P. Mali-Infected Malus × Domestica but not in the Insect Vector C. Picta

**Figure 2:**
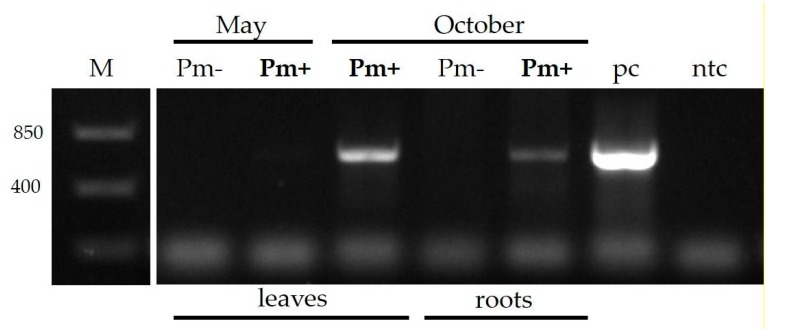
Expression of *pme*2 (CAP18323.1) in ’*Candidatus* Phytoplasma mali’ (P. mali)-infected *Malus × domestica*. Transcripts of *pme*2 were detected by PCR using cDNA from *Malus × domestica* infected with P. mali. A discrete band of the size indicative for the *pme*2 transcript was detected in P. mali-infected (**Pm+**) but not in non-infected (Pm–) leaves and roots harvested in October. DNA derived from an infected *Malus × domestica* served as a positive control (pc) and water as the non-template control (ntc).

Subsequently, it was analyzed whether *atp_00136* was expressed in apple trees infected with P. mali. Leaf and root samples of P. mali-infected and non-infected *Malus × domestica* cv. “Golden Delicious” trees were taken in May and October. Expression of *atp_00136* was analyzed with *atp_00136*-specific primers and *Malus × domestica* cDNA derived from mRNA. Expression of *atp_00136* was confirmed in P. mali-infected leaf and root tissue by the detection of distinct amplicons at the expected size in the respective samples (Figure 2). 

Using quantitative PCR (qPCR) the expression levels of *atp_00136* and P. mali in the samples were quantified. The results show that *atp_00136* is only expressed in tissue colonized by P. mali (Table 1). Since identified expressed genes were named in a chronological manner, *atp_00136* was named “Protein in *Malus* Expressed 2” (*pme2*) based on the general recommendations for bacterial gene nomenclature [28].

To analyze if *pme2* was expressed in the transmitting insect vectors during infection, three P. mali-infected *C. picta* individuals were analyzed for the expression of the potential effector. In the RNA/cDNA of all infected individuals, P. mali-specific transcripts of the ribosomal protein *rpl22* were detected, but expression of *pme2* was not detectable.

### 2.3. Genetic Variability of *Pme2*

**Figure 3:**
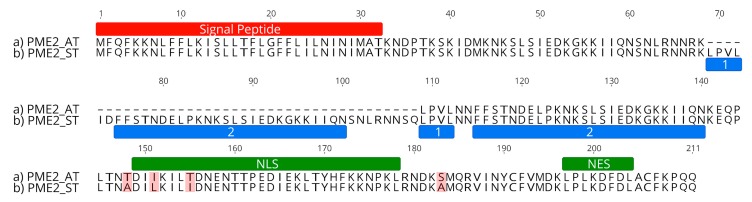
Sequence comparison of PME2_ST_ and PME2_AT_. The protein variants (**a**) PME2_AT_ and (**b**) PME2_ST_ contain the same *N*-terminal signal peptide sequence (red). PME2_ST_ (**b**) contains a duplicated amino acid stretch (the replicative sequences 1 and 2; marked in blue) of a partial sequence also present in PME2_AT_ (**a**). Both variants show slight differences in and directly before the nuclear localization signal sequences (NLS, green). The nuclear export signal sequence (NES, green) is identical in both protein variants. Amino acid differences of PME2_ST_ to the PME2_AT_ variant are shown in black, whereas similarities are shown in grey. Graphs were generated with Geneious Prime 2018 version 11.1.4.

Cloned amplicon sequencing revealed that the prevalent variant of *pme2* from infected trees in South Tyrol (North-East Italy) differs compared to the *pme2* sequence of the P. mali AT strain from Germany [19]. In a total of 20 samples from naturally infected apple trees in the regions Burggraviato and Val Venosta, a prevalent, conserved sequence of *pme2* was identified (*pme2*_ST_; accession number MN224214). This conserved variant exhibits a single nucleotide polymorphism (SNP) in the sequence stretch before the NLS, and two SNPs within and one SNP after the NLS compared to the AT strain (Figure 3). All four SNPs in the *pme2*_ST_ variant lead to nonsynonymous missense substitutions at the protein level as compared to the *pme2* sequence published previously [19] (*pme2*_AT_). The NLS of *pme2*_ST_ has a slightly higher prediction score than the NLS of *pme2*_AT_. The most striking difference between *pme2*_AT_ and *pme2*_ST_ is a stretch of 120 bp in *pme2*_ST_ which is absent in *pme2*_AT_. This stretch is a partial duplication of a fragment also present in *pme2*_AT_ (Figure 3). In three *Malus × domestica* samples, a very sporadic sequence of *pme2* could be detected that did not contain the *pme2*_ST_ characteristic sequence duplication but showed strong sequence similarity to *pme2*_AT_. The sporadic sequence contains six SNPs at positions 218 (A > T), 220 (A > G), 322 (A > G), 331 (A > C), 344 (C < T), and 427 (T > G) that lead to nonsynonymous missense mutations (accession number MN224215) compared to *pme2*_AT_. However, in the trees in which these very sporadic *pme2* sequences were found, *pme2*_ST_ could also be detected, indicating the presence of a mixed population of different P. mali strains.

### 2.4. PME2_ST_ and PME2_AT_ Translocate to the Nucleus of *Nicotiana* spp. Protoplasts

**Figure 4:**
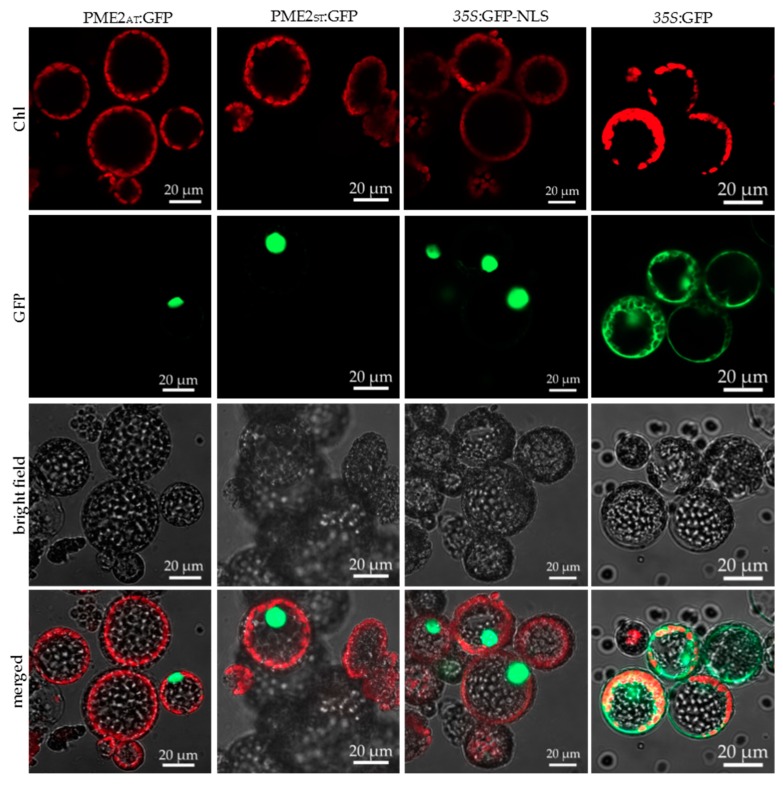
PME2_ST_ and PME2_AT_ are translocated to the nucleus of mesophyll protoplasts. Mesophyll protoplasts of *Nicotiana benthamiana* were transformed with the plasmid pGGZ001 encoding C-terminal GFP-tagged PME2_ST_ (first column), PME2_AT_ (second column), GFP *N*-terminally fused to a NLS sequence (third column), or GFP only as a control for nuclear localization (fourth column). Expression of the transgenes was under the control of a *35S* promoter. The upper panel shows autofluorescence of chloroplasts (Chl), the second panel the signal derived from the GFP, and the third panel the bright field image and the last panel an overlay of all images (merged). Microscopic analysis was performed with a Zeiss LSM 800. Corresponding images after expression of mCherry-tagged PME2 and of use of *Nicotiana occidentalis* mesophyll protoplasts are presented in Figures S1–S3. Bars represent 20 µm.

To identify the subcellular localization of the PME2 protein in the plant cell, mesophyll protoplasts of *Nicotiana occidentalis* and *N. benthamiana* were transformed, with expression vectors coding for PME2_AT_ and PME2_ST_ tagged with GFP or mCherry-fluorescent protein to allow subcellular tracking. The *N*-terminal signal part was not considered for these studies, since it is removed from the processed, mature CAP18323.1 protein. *N. occidentalis* and *N. benthamiana* can be infected with P. mali. Upon infection, both *Nicotiana* species show disease-specific symptoms and are thus appropriate model plants for P. mali effector studies [15,29]. Confocal microscopy analysis revealed that overexpressed PME2_AT_ and PME2_ST_ are translocated to the nucleus of *Nicotiana* spp. protoplasts. This translocation was independent of the used tag and *Nicotiana* species (Figure 4 and Figures S1–S3). The in vivo results therefore confirm the in silico prediction that PME2_ST_ and PME2_AT_ are translocated to the nucleus of potential host cells.

A leaf infiltration assay using *Agrobacterium* strain EHA105 transformed with PME2 encoding expression vectors did not result in detectable expression or phenotypic alterations of either PME2_AT_ or PME2_ST_ in both *Nicotiana* species. Nonetheless, positive controls expressing the fluorophore tag only and leaves infiltrated with the P. mali SAP11-like effector protein ATP_00189 [13] as control showed strong signals (Figure S5), indicating that PME2 expression might be somehow blocked or is immediately degraded by the plant.

### 2.5. PME2_ST_ but not PME2_AT_ Affect Cell Integrity of *Nicotiana* spp. Protoplasts

**Figure 5:**
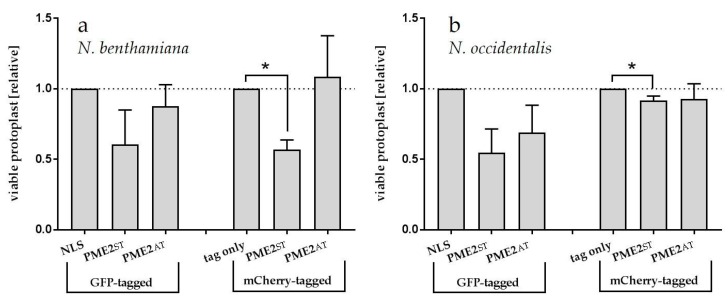
PME2_ST_ overexpression reduces viability of (**a**) *N. benthamiana* and (**b**) *N. occidentalis* mesophyll protoplasts. For each assay, 20,000 mesophyll protoplasts were transformed with the plasmid pGGZ001 encoding PME2_ST_, PME2_AT_ (tagged with GFP or mCherry), the GFP-tagged control for nuclear localization (NLS), or the mCherry tag (tag only) and viable protoplasts were counted. Overexpression of the transgenes was under the control of a *35S* promoter. Data represent the mean viability +/– SE of 3–4 independent experiments. The respective control (NLS or tag only) was set at 1 to allow comparison between different experiments. Differences between the groups were determined applying a one way-ANOVA analysis. Significant differences (*p* < 0.05) between groups are indicated with an asterisk (*).

**Figure 6:**
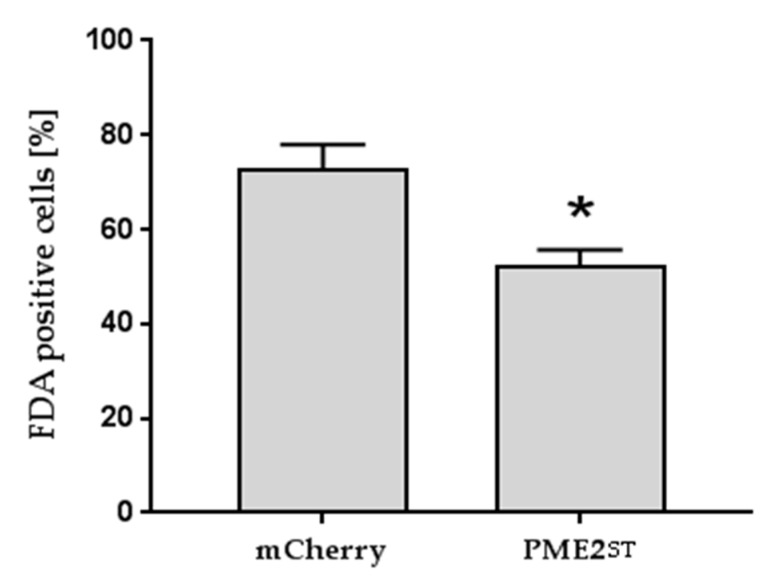
PME2_ST_ overexpression reduces viability of *N. benthamiana* mesophyll protoplasts. Mesophyll protoplasts were transformed with the plasmid pGGZ001 encoding mCherry-tagged PME2_ST_ (PME_ST_) or the mCherry tag only (mCherry) and stained with fluorescein diacetate (FDA) to detect viable cells. Data represent the mean percentage of FDA-positive stained cells +/– SE (*n* = 3). The statistical difference between the two groups was determined by using a Student’s *t*-test and is indicated with an asterisk (*p < 0.05).

Protoplasts transformed with the PME2_ST_ expression vector often showed shrinkage, and only about 50% of the *N. benthamiana* protoplasts were viable 20 h post-transformation compared to the transformation control expressing the fluorophore only or a GFP with NLS (Figure 5a). The shrunk cells lysed and only the remaining cell debris was microscopically detectable (Figure 4). The effect on protoplast integrity was observed in protoplasts expressing PME2_ST_:GFP and PME2_ST_:mCherry, and thus was independent of the fluorophore used as a tag for microscopic analyses. Similar results were obtained using *N. occidentalis* as the heterologous PME2_ST_ expression system. The mCherry-tagged PME2_ST_ induced a weak but significant reduction of viability in *N. occidentalis* protoplasts (Figure 5b). The GFP-tagged PME2_ST_ showed the same tendency but to a stronger extent, i.e., it reduced cell viability by about 50%, which is similar to the effect seen in *N. benthamiana* protoplasts. Cell viability stain with fluorescein diacetate (FDA) showed similar results, i.e., that *N. benthamiana* protoplasts transformed with the PME2_ST_-expressing vector showed a significantly reduced viability (Figure 6). Shrunk cells were positive for propidium iodide (PI) staining (Figure S4), indicating that these cells were dead.

Interestingly, PME2_AT_ did not have an effect on protoplast integrity in *N. benthamiana* nor in *N. occidentalis* protoplasts (Figure 5).

### 2.6. A Yeast Two-Hybrid Screen Was Unsuitable for the Elucidation of PME2_ST_ Function

**Figure 7:**
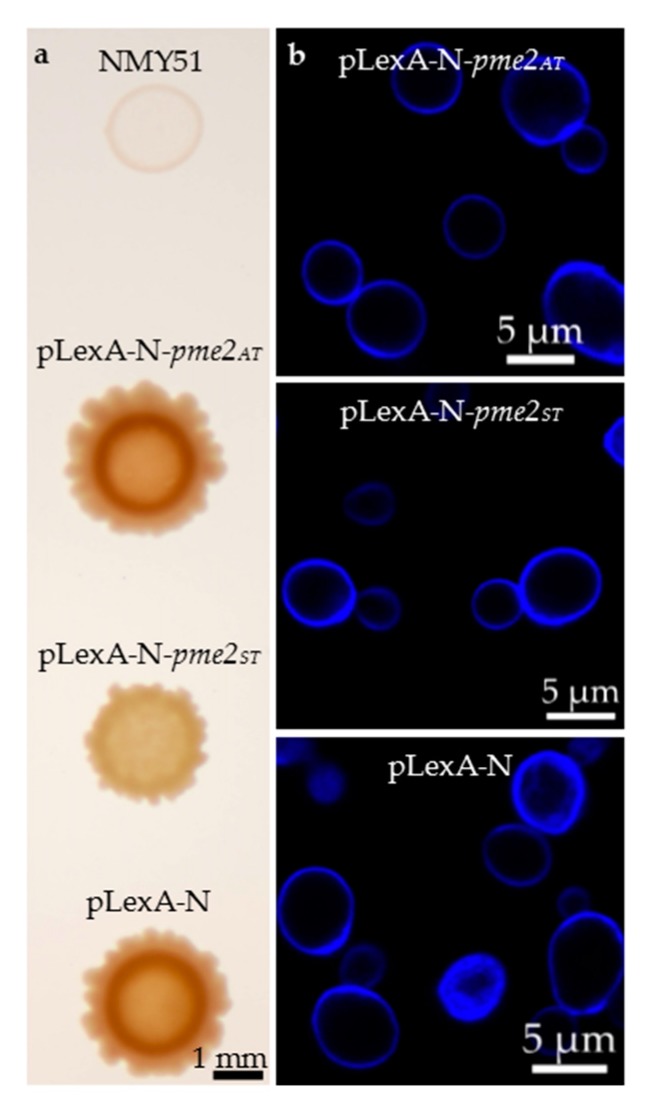
PME2_ST_ overexpression in the yeast reporter strain NMY51 leads to macroscopic aberrations. *Saccharomyces cerevisiae* strain NMY51 was transformed with the yeast two-hybrid (Y2H) bait vector pLexA-N, which encodes tryptophan auxotrophy, expressing PME2_AT_, PME2_ST_, or the empty vector only, and drop-plated onto SD-trp plates. In comparison to the empty pLexA-N vector and the vector expressing PME_AT_, colonies expressing PME_ST_ showed reduced growth and remained white (**a**). Yeast cells stained with calcofluor white did not show any phenotypic differences on single-cell level (**b**). Calcofluor white fluorescence was visualized on a confocal microscope (LSM 800, Carl Zeiss AG, Oberkochen, Germany).

Upon expression of PME2_ST_, the yeast reporter strain *Saccharomyces cerevisiae* NMY51 showed several macroscopic aberrations in colony growth (Figure 7a). However, at the microscopic level when visualizing the yeast cell wall with calcofluor white, no phenotypic differences between yeast cells expressing PME2_AT_, PME2_ST_, and empty bait vector pLexA-N could be detected (Figure 7b). Considering the effect of mere PME2_ST_ expression on growth of the yeast reporter strain, the relevance of any identified interaction in a yeast two-hybrid screen remains highly questionable and the assay was therefore not performed.

## 3. Discussion

The results of this study show that PME2_ST_ (a variant of CAP18323.1 previously annotated as “conserved hypothetical protein”) affects plant cell integrity. Based on our findings and the definition that effectors are secreted pathogen proteins altering host-cell structure and function [30], we propose defining PME2 as a phytoplasmal effector. Interestingly, two different variants of PME2 were identified and both variants translocate to the nucleus of plant cells, but only the newly described regional variant PME2_ST_ subsequently affects protoplast integrity. The small size of about (at a maximum) 21 kDa (PME2_ST_: 21 kDa and PME2_AT_: 16 kDa; both considering the mature protein without the signal peptide) indicates that PME2 can be translocated from the phloem and target adjacent tissues or be distributed systemically in the plant [3]. Subcellular localization using microscopy requires the use of fluorescent tags that are attached to the protein of interest. Tagging can affect subcellular localization of the protein; however, we used two different tags (GFP and mCherry) to analyze whether tagging influences the target localization. In cells expressing the tag only, a localization of the fluorescent signal in the cytoplasm could be observed. PME2 was localized only in the nucleus and since no signal was visible in other cell compartments, it can be assumed that the observed localization is effector-mediated (see also [31]). Only protoplasts transformed with PME2_ST_ showed significant cell disruption as indirectly quantified by counting the remaining viable cells and FDA staining of the protoplasts. Shrunk cells were positive for the PI stain but did not show a GFP signal. The lack of the GFP-signal might be caused by a disruption of the nucleus, protein degradation, and/or leakage of the signal into the surrounding medium. Cells expressing PME2_ST_ were intact, indicating that the effector either exhibits a dose-dependent or delayed effect on cell integrity.

Both variants of PME2 contain an *N*-terminal signal peptide, a nuclear localization signal (NLS), and a nuclear export signal (NES). It is a common feature of nuclear proteins to contain both NLS and NES and these signals coordinate the translocation of the protein between nucleus and cytoplasm [32]. Nuclear targeting of proteins containing a classical NLS is mediated by the importin α/β heterodimer through NLS-dependent binding to the importin α subunit and importin β–mediated attachment to the nuclear pore complex [33,34]. The SNPs in the NLS region of PME2_ST_ lead to a (slightly) higher sequence-based NLS prediction; thus, the differences might show a stronger translocation to the nucleus. The NES signal (which indicates that shuttling of PME2 between nucleus and cytoplasm might occur) is the same in both variants. Even though nucleocytoplasmic distribution is predicted, PME2 was only detected in the nucleus. Many proteins containing NLS and NES appear to be localized in the nucleus because the rate of import to the nucleus is higher than the rate of export to the cytoplasm [35]. It remains thus unclear if PME2 is strictly limited to the nucleus or if a constant shuttling between nucleus and cytosol occurs.

Bacterial effectors that translocate to the nucleus, the so-called nuclear effectors, can affect master switches of the host immune machinery or alter host transcription to the benefit of the pathogen [31]. Effectors from different phytoplasma species target plant–host transcription factors or affect gene expression on the transcriptional level to alter the host metabolism to their own benefit [4,12,13,36,37]. However, none of these effectors have yet been reported to exhibit such detrimental effects during in planta expression. The effector protein BR1 of the phloem colonizing squash leaf curl geminivirus shuttles between the cytoplasm and the nucleus of protoplasts [38]. Upon binding to the second movement protein BL1, BR1 shuttles to the cytoplasma [39] and the concerted action between BR1 and BL1 mediates cell-to-cell movement of the virus within the phloem and to adjacent cells [35,38,40,41]. To unravel BR1 function it was necessary to identify its interaction partner, a general approach to investigate effector function. Yeast two-hybrid (Y2H) screens have been successfully applied to determine phytoplasmal effector targets on the molecular level [10,13]. These screens allow the screening of a protein of interest (effector) against a library containing hundreds of thousands of different potential interaction partners of a certain host species [42,43]. Successful interaction is monitored by a genetic reporter system that complements certain auxotrophies in the recombinant yeast reporter strain. However, a Y2H with PME2_ST_ is not suitable since PME2_ST_ expression strongly affected the Y2H yeast reporter strain. This effect on yeast cells further supports the finding that PME2_ST_ exhibits a strong effect not only on plant, but also on yeast cells, even though the latter do not have relevance as phytoplasma host cells. Since PME2_ST_ exhibits such a strong effect on the expressing host and non-host cells, alternative approaches must be applied to unravel its molecular function. *Nicotiana* spp. leaf infiltration assays with recombinant *Agrobacterium* strains expressing PME2 failed. It remains furthermore elusive as to whether PME2 exhibits effects on the host plant phenotype. Considering the PME2_ST_ effects on protoplasts it can be assumed that a systemic overexpression would lead to overwhelming deleterious effects in transgenic plants that express this effector. The results show that PME2 is expressed in roots and leaves of infected *Malus × domestica*, but not in infected individuals of its insect vector *C. picta*, underlining the hypothesis that PME2 plays a role as an effector protein in plant cells. However, it needs further clarification if expression is fine-tuned in a tempo-spatial manner in the plant host.

A neatly coordinated and local expression during infection might have very local effects and might not lead to cell disruption as seen in heterologous overexpression experiments. It is hypothesized that phytoplasma are able to degrade plant cell walls or generate holes in plant cell membranes to expedite cell-to-cell effector translocation [4]. Infection with P. mali induces cytochemical modifications and injuries of the affected phloem cells [44,45]. It is speculated that plasma membrane integrity is affected by until-now unknown P. mali effector(s) and that plasma membrane disruption is involved in the observed phloem damage induced by virulent P. mali strains [45]. However, since molecular indications are lacking, interpretation of the mode of PME2 action remains speculative. Subsequent approaches to analyze PME2 function should comprise assays that do not depend on functional living cells.

Since PME2 is translocated to the nucleus it is possible that it directly targets the host DNA by mimicking DNA regulatory elements, such as transcription factors or repressors. Some plant pathogen effectors bind host DNA and thus modulate gene expression [46,47]. An example of these effectors are TAL effectors of the plant pathogen *Xanthomonas*. TAL effectors bind promoter elements and regulate plant host expression to the benefit of the pathogen [48,49,50,51]. The effector AvrBs3 of *Xanthomonas* translocates to the nucleus where it acts as a transcription factor and affects the size of mesophyll cells [52]. Bioinformatic prediction and sequence comparison did not indicate that PME2 has similarity with currently known transcription factors or other gene expression regulating factors in plants.

Both P. mali strains from which the two different PME2 variants were derived cause infection and typical disease symptoms in *Malus × domestica*. Thus, the effect of PME2_ST_ on cell integrity seems to be dispensable for infection and symptom development but might affect strain virulence. However, a direct comparison between the two strains regarding their virulence is missing. It might also be possible that another effector of P. mali strain AT (unknown at the time of this research), mimics and thus complements the function that PME2_AT_ is lacking.

Some P. mali strains strongly differ regarding their virulence potential in *Malus × domestica* and several studies addressed the genetic identification of virulence factors or certain genetic determinants that account for these differences [17,18,53,54,55]. Since phytoplasma cannot be genetically manipulated, determining the importance of an effector during infection often involves tortuous experimental paths. In this study we provide the first characterization of the P. mali effector PME2 and its effect on cells of potential plant hosts. We report an interesting difference between two variants of PME2 that occur in Italy and Germany, claiming that further full genomic sequence analysis is required to better understand how P. mali manipulates its host on the molecular level.

## 4. Materials and Methods

### 4.1. Verification of *Pme2* Expression in *Malus × Domestica* and *C. Picta*

For the verification of *pme2* expression by PCR in infected apple root and leaf samples RNA was extracted from the plant tissue as described in [13]. Extracted RNA was subjected to DNase treatment using DNAfree Turbo reagent (Ambion, Austin, TX, USA) and cDNA synthesis was performed using the SuperScript™ VILO™ cDNA Synthesis Kit (Invitrogen, Waltham, MA, USA). The generated cDNA was diluted 1:200 in nuclease free water and cDNA integrity was checked in all samples by performing a control PCR targeting the house-keeping gene transcript putative tip41-like family (transcript identifier: Mdo.1349) using the primers 5’-ACATGCCGGAGATGGTGTTTGG-3’ (forward) and 5’-ACTTCCAGAGTACGGCGTTGTG-3’ (reverse). Contamination with genomic DNA was checked by performing a PCR with primers amplifying a fragment within the non-coding region trnL of chloroplast DNA using the primers B49317 and A49855 [56]. No DNA contamination was detected in any of the cDNA samples, and the amplification of the putative tip41-like transcript fragment was positive, thus confirming the integrity of the generated cDNA. PCR reactions to verify *pme2* expression were set up in a total reaction volume of 10 µL, using 2 µL of diluted cDNA (1:200) as template, 0.05 µL GoTaq^®^ DNA Polymerase (Promega, Madison, WI, USA), 2 µL of 5X Green GoTaq^®^ Reaction Buffer (Promega, Madison, WI, USA), 0.2 µL dNTP-mix (40 mM), 1 µL of forward primer ATP00136_forw_EcoRI (10 µM, 5’-CCCCCCGAATTCATGTTTCAATTTAAAAAAAATTTA-3’), and 1 µL of reverse primer ATP00136_rev_SalI (10 µM, 5’-CCCCCCGTCGACATTATTACTGTTGAGGTTTAA-3’). Cycling conditions were applied as follows: 95 °C for 5 min followed by 40 cycles of 95 °C for 1 min, 44.9 °C for 1 min, 72 °C for 1 min, and a final elongation step at 72 °C for 5 min. PCR products were visualized on 1% agarose gel. Additionally, *pme2* expression level was detected by qPCR based on SYBR-Green chemistry using the primer pair ATP00136_GW_fwd (5’-CACCATGACGAAAAATGATCCAACAAA-3’)/ATP00136_nostopp_rev (5’-CTGTTGAGGTTTAAAACAT-3’) in a total reaction volume of 20 µL using 4.0 µL of diluted cDNA (1:200) as a template together with 10.0 µL 2× SYBR FAST qPCR Kit Master Mix (Kapa Biosystems/αmann-La Roche, Basel, Switzerland), 1.0 µL of each primer (10 µM), and 4.0 µL of nuclease free water. qPCR conditions were as follows: an initial denaturation step at 95 °C for 20 s followed by 34 cycles of 95 °C for 3 s and 60 °C for 30 s and a melting curve ramp from 65 to 95 °C, at increments of 0.5 °C every 5 s (CFX384 Touch Real-Time PCR Detection System; BioRad, Hercules, CA, USA). Data analysis was performed using the CFX ManagerTM software (BioRad, Hercules, CA, USA).

To control whether *pme2* is expressed in infected individuals of the insect vector *C. picta*, RNA of six potentially infected and two uninfected F1 individuals was extracted with the ZR Tissue & Insect RNA MicroPrep^TM^ kit (ZymoResearch, Irvine, CA, USA) according to the manufacturer’s instructions. Extracted RNA was subjected to DNase treatment using DNAfree Turbo reagent (Ambion, Austin, TX, USA) and RNA integrity was controlled with an RNA ScreenTape on a TapeStation 2200 (both Agilent, Santa Clara, CA, USA). cDNA was synthesized with the iScript^TM^ cDNA Synthesis Kit (BioRad, Hercules, CA, USA). Together with the cDNA synthesis a control was performed lacking the reverse transcriptase (-RT). Here, 2 µL of diluted cDNA (1:200) were used as template in a total qPCR reaction volume of 10 µL, together with 5 µL 2× SYBR FAST qPCR Kit Master Mix (Kapa Biosystems/Hoffmann-La Roche, Basel, Switzerland), 2 µL of nuclease free water, and 0.5 µL of forward and reverse primer (10 µM). The primer combination qPSY-WG-F and qPSY-WG-R, targeting the species-specific *wingless* gene [57], was used to determine cDNA integrity. P. mali infection was detected in three of the six individuals with primer pair rpAP15f-mod and rpAP15r3, targeting the ribosomal protein gene *rpl22* [58]. *Pme2* expression was checked with primer pair ATP00136_GW_fwd and ATP00136_nostopp_rev using the same qPCR conditions as described for the qPCR detection in *Malus × domestica* leaf samples.

### 4.2. Amplification, Subcloning, and Sequencing of atp_00136 

DNA was purified from leaves from P. mali infected *Malus x domestica* cv Golden Delicious trees (10 trees from Burggraviato and 10 trees from Val Venosta) using the DNeasy Plant Mini kit (Qiagen, Hilden, Germany) following the manufacturer’s instructions. DNA was diluted 1:10 in water and 2 µL template were used in a total PCR reaction volume of 50 µL as follows: *atp_00136* was amplified using 0.02 U/µL Phusion High-Fidelity DNA Polymerase (Thermo Fisher Scientific, Waltham, MA, USA) using HF-buffer supplied by the manufacturer, 400 µM dNTPs, and 0.5 µM of each primer (forward: 5’-CCCCCCGAATTCATGTTTCAATTTAAAAAAAATTTA-3’; reverse: 5’-CCCCCCGTCGACATTATTACTGTTGAGGTTTAA-3’). DNA was denatured at 98 °C for 30 s followed by 30 cycles of denaturation for 10 s at 98 °C, amplification for 30 s at 49.3 °C, and elongation at 72 °C for 30 s. The PCR was finalized by a terminal elongation step at 72 °C for 5 min. The PCR product was purified using the Illustra GFX PCR DNA and Gel Band Purification Kit (GE Healthcare, Chicago, IL, USA) and 1 µg of purified PCR product was digested with 4 U EcoRI and SalI following the manufacturer’s instructions (Thermo Fisher Scientific, Waltham, MA, USA), ligated into equally digested pUC19 using T4-Ligase (Thermo Fisher Scientific, Waltham, MA, USA) and transformed into MegaX DH10B™ T1R cells (Life Technologies, Carlsbad, CA, USA). At least five clones from each tree were sequenced with pUC19 specific primers (GATC Biotech, Constance, Germany) and analyzed to see different variants of the gene indicating a mixed infection.

### 4.3. Subcloning of *Pme2* into GreenGate Expression Vectors

The genes *pme2*_ST_ and *pme2*_AT_ were subcloned into the GreenGate-entry module pGGC000 [59] using the primer pair ATP00136pP_CBsaI_fw (5’-AACAGGTCTCAGGCTCCATGACGAAAAATGATCCAACAAA-3’) and ATP00136pP_DBsaI_rv (5’-AACAGGTCTCACTGACTGTTGAGGTTTAAAACAT-3’). Using different components from the GreenGate-kit plant, transformation constructs coding for *pme2_AT_-linker-GFP* or *pme2_AT_-linker-mCherry* and *pme2_ST_-linker-GFP* or *pme2_ST_-linker-mCherry*, driven by the *35S* promoter and flanked at the 3’-end by the *RBCS* terminator, including kanamycin as the plant resistance marker, were designed. The following modules were assembled by GreenGate reaction in a total volume of 15 µL: 150 ng pGGA004 (*35S*), 150 ng pGGB003 (B-dummy), 150 ng pGGC000-*pme2_AT_* or pGGC00-*pme2_ST_*, 150 ng pGGD001 (*linker-GFP*) or pGGD003 (*linker-mCherry*), 150 ng pGGE001 (*RBCS*), 150 ng pGGF007 (*pNOS*:*Kan^R^*:*tNOS*), and 100 ng pGGZ001 (empty destination vector). Subsequently, 1.5 µL 10× CutSmart Buffer (New England Biolab, Ipswich, MA, USA), 1.5 µL ATP (10 mM), 1.0 µL T4 DNA Ligase (5 u/µL) (Thermo Fisher Scientific, Waltham, MA, USA), and 1.0 µL BsaI-HF^®^v2 (20,000 u/mL) (New England Biolab, Ipswich, MA, USA) were added to the module mixture, and 30 cycles of 2 min at 37 °C and 2 min at 16 °C each, followed by 50 °C for 5 min and 80 °C for 5 min were performed. Subsequently, 5 µL of the reaction mixture were used for heat-shock transformation of *ccdB*-sensitive One Shot^®^ TOP10 chemically competent *Escherichia coli* (Invitrogen, Carlsbad, CA, USA). For the assembly of positive controls, the modules pGGC012 (*GFP-NLS*) or pGGC014 (*GFP*) or pGGC015 (*mCherry*) were used instead of the above mentioned pGGC000 modules. The correct assembly of the plant transformation constructs was confirmed by sequencing. Plasmid-DNA for protoplast transformation was obtained as described elsewhere [60], using the NucleoSnap^®^ Plasmid Midi preparation kit (Macherey-Nagel, Düren, Germany) and PEG precipitation.

### 4.4. Protoplast Isolation and Transformation

Protoplasts of *N. benthamiana* and *N. occidentalis* were isolated from four- to five-week-old plants, cultivated under long photoperiod conditions (16 h/8 h, 24 °C/22 °C, 70% rH) and transformed as described in [60] using 10 µg plasmid-DNA per 20,000 protoplasts. After 18 h, at least 100 protoplasts of each transformation were checked for the occurrence of GFP or mCherry-fluorescence using a confocal laser scanning microscope (LSM800, Zeiss, Oberkochen, Germany) with an excitation wavelength of 488 nm for GFP and 561 nm for mCherry. The detection wavelength of GFP was set between 410 nm and 575 nm and of mCherry between 575 nm and 650 nm. Autofluorescence of chlorophyll was detected between 650 nm and 700 nm. After 20 h the number of intact protoplasts/mL was determined by counting in a Fuchs-Rosenthal chamber. Protoplast transformation and viability determination was repeated independently four times.

Only experiments in which at least 20% of the protoplasts in the control setup were viable after transformation were considered for further evaluation. Significant outliers were removed from the data set using the GraphPad QuickCalcs Outlier calculator online tool (https://www.graphpad.com/quickcalcs/Grubbs1.cfm; status of information 16th September 2019). Greisser Greenhouse correction on raw data and one-way-ANOVA with a Tukey Posttest were performed to analyze statistical differences between groups (GraphPad Prism 7.01., GraphPad Software, San Diego, CA, USA). To allow a better visual comparison, data were normalized to each respective control, which was set to 1.

Additionally, protoplast viability was visualized by propidium iodide (PI) and counted by fluorescein diacetate (FDA) staining in three independent repetitions. For the first, 20 µL of protoplasts transformed with GFP tagged expression vectors were mixed with 20 µL of PI solution (10 µg/mL PI in 0.65 M mannitol). FDA staining was done according to [61] using 20 µL of protoplasts transformed with either mCherry tagged PME2_AT_ expression vectors or a vector expressing only mCherry and 20 µL of FDA solution (0.1 mg/mL FDA in 0.65 M mannitol). Fluorescence of PI, mCherry, and GFP was recorded using a LSM800 confocal laser scanning microscope (Zeiss, Oberkochen, Germany) with excitation and detection wavelengths for GFP and mCherry as described above and for PI excitation at 561 nm and detection between 560 nm and 640 nm.

### 4.5. Nicotiana spp. Leaf Infiltration

For subcellular localization of PME2, the two GreenGate expression vectors, as well as GFP and GFP-NLS expression vectors as positive controls, were subcloned by electroporation into *Agrobacterium tumefaciens* strain EHA105. As an additional control, we subcloned a GreenGate expression vector expressing the SAP11-like P. mali effector protein ATP_00189 [13] with an *N*-terminal fused GFP tag into *A. tumefaciens* strain EHA105. The transgenic *A. tumefaciens* clones were cultured for 2 days at 28 °C in liquid selective LB medium. Subsequently, 0.5 OD/mL were resuspended in infiltration medium (10 mM MgCl_2_, 10 mM MES, 200 µM acetosyringone, pH 5.7) and infiltrated with a blunt syringe into leaves from four- to five-week-old *N. occidentalis* and *N. benthamiana*. Fluorescence was recorded after 48 h and 72 h using the confocal laser scanning microscope (LSM800, Zeiss, Oberkochen, Germany) with excitation for GFP at 488 nm and detection between 410 nm and 546 nm and excitation for mCherry at 561 nm and detection between 562 and 624 nm.

### 4.6. Expression in Yeast

For a potential Y2H, *pme2_AT_* and *pme2_ST_* were subcloned into bait-vector pLexA-N as described in [13,62] with primer pair ATP00136_forw_EcoRI/ATP00136_rev_SalI. The bait-plasmids pLexA-N-*pme2_ST_* and pLexA-N-*pme2_AT_* were transformed into *S. cerevisiae* strain NMY51. Growth aberrations of yeast colonies on selective SD-trp plates were observed and recorded by photographing.

For calcofluor white staining, yeast cells were grown overnight in SD-trp liquid media. Subsequently, 2 mL of the overnight culture were centrifuged, supernatant removed, and the cells resuspended in clear phosphate-buffered saline (PBS) buffer. Then, 10 µL of a 5 mM calcofluor white solution (Biotium, Fremont, California) were added to the cell suspension and incubated for 20 min at room temperature. The yeast cell wall was visualized by a confocal laser scanning microscope (LSM800, Zeiss, Oberkochen, Germany) with excitation at 405 nm and detection wavelength between 400 nm and 560 nm.

## 5. Conclusions

In this study we identified and characterized the novel P. mali effector protein PME2. This effector contains an NLS and an NES sequence and translocates to the nucleus of *N. benthamiana* mesophyll protoplasts. Two naturally occurring genetic variants of PME2, namely PME2_ST_ and PME2_AT_, differ regarding their ability to induce cellular modifications in yeast and plant cells. When overexpressed, the variant PME2_ST_ affects yeast growth and reduces the viability of *Nicotiana* spp. mesophyll protoplasts. These findings indicate that PME2 might play a role for P. mali virulence in plants. Despite the similarities between both PME2 variants, this effect was not observed in yeast or protoplasts expressing PME2_AT_. The results of our study show for the first time that a phytoplasmal effector causes detrimental effects when overexpressed in protoplasts.

## Figures and Tables

**Table 1:** Detection of *atp_00136* in cDNA samples from infected and non-infected leaf tissue from May and October 2011. In May phytoplasma were only detectable in the roots but not in the leaves. *atp_00136* was only detectable in P. mali-infected and colonized tissue. Cq values are given as the mean value of three repeated qPCR runs.

Month	Status	Pool	cDNA Integrity (*tip41*)	Phytoplasma (*16S*)	*atp_00136*
May	non-infected	1	26.38	N/A	N/A
		2	26.38	N/A	N/A
Oct	non-infected	3	26.58	N/A	N/A
		2	26.59	N/A	N/A
		3	26.58	N/A	N/A
May	infected	1	26.56	N/A	N/A
		2	26.53	N/A	N/A
		3	26.61	N/A	N/A
Oct	infected	1	26.71	23.67	28.00
		2	26.44	23.18	27.66
		3	26.48	23.34	28.34

## Supplementary Materials

Supplementary materials can be found at https://www.mdpi.com/1422-0067/20/18/4613/s1.

## Abbreviations

AP	Apple proliferation
AY-WB	Aster Yellow Witches’ Broom
*C. picta*	*Cacopsylla picta*
FDA	Fluorescein diacetate
GFP	Green fluorescent protein
*N.*	*Nicotiana*
NES	Nuclear export signal
NLS	Nuclear localization signal
PI	Propidium iodide
P. mali	*Candidatus* Phytoplasma mali
PME2	Protein in Malus Expressed 2
qPCR	quantitative PCR
SNP	Single nucleotide polymorphism
Y2H	Yeast two-hybrid
