# Supplementary material for: A Novel Effector Protein of Apple Proliferation Phytoplasma Disrupts Cell Integrity of Nicotiana spp. Protoplasts
Source: Int J Mol Sci. 2019 Sep 18;20(18):4613. doi: 10.3390/ijms20184613 (PMC6770106; doi:10.3390/ijms20184613)
Supplement: Supplementary file 1 [file ijms-20-04613-s001.pdf]

International Journal of *Molecular Sciences*

MDPI

Supplementary Materials

A Novel Effector Protein of Apple Proliferation Phytoplasma Disrupts Cell Integrity of *Nicotiana* spp. Protoplasts.

Cecilia Mittelberger[1], Hagen Stellmach[2], Bettina Hause[2], Christine Kerschbamer[1], Katja Schlink[1], Thomas Letschka[1] and Katrin Janik[1*]

[1]Functional Genomics, Laimburg Research Centre, Auer/Ora (BZ), Italy
[2] Jasmonate Function & Mycorrhiza, Leibniz Institute of Plant Biochemistry, Halle, Germany

*Correspondence: katrin.janik@laimburg.it

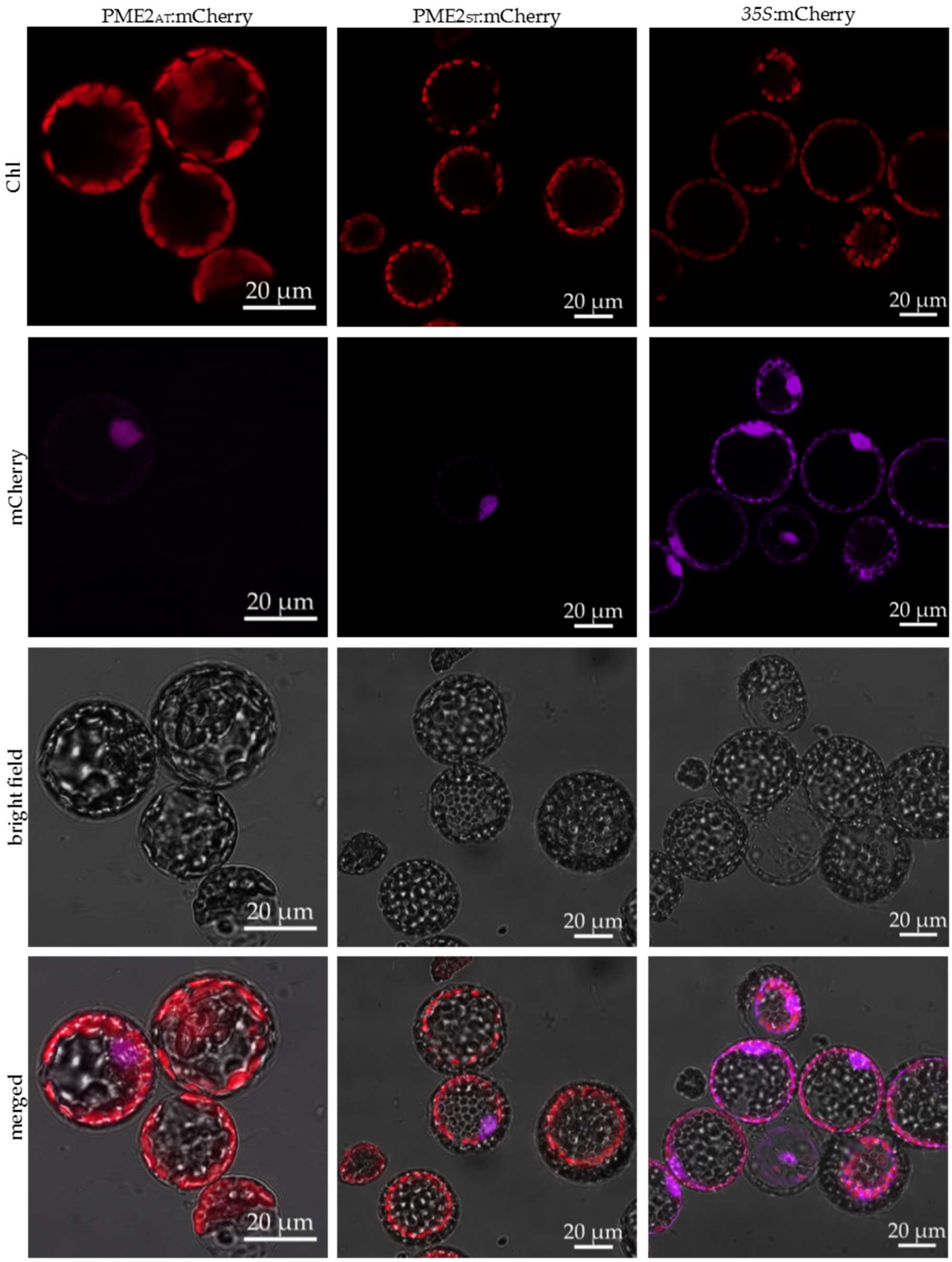

Figure S1. PME2ST and PME2AT are translocated to the nucleus of mesophyll protoplasts. Mesophyll protoplasts of *Nicotiana benthamiana* were transformed with the plasmid pGGZ001 encoding C-terminal mCherry-tagged PME2ST (left column), PME2AT (middle column) or mCherry (right column) as a control. Expression of the transgenes was under the control of a *35S* promoter. The upper panel shows autofluorescence of chloroplasts (Chl), the second panel the signal derived from mCherry, the third panel the bright field image and the last panel an overlay all images (merged). Protoplasts transformed with the PME2ST expressing vector showed frequently shrinkage and cell disruption (arrow in the middle column). Microscopic analysis was performed with a Zeiss LSM 800.

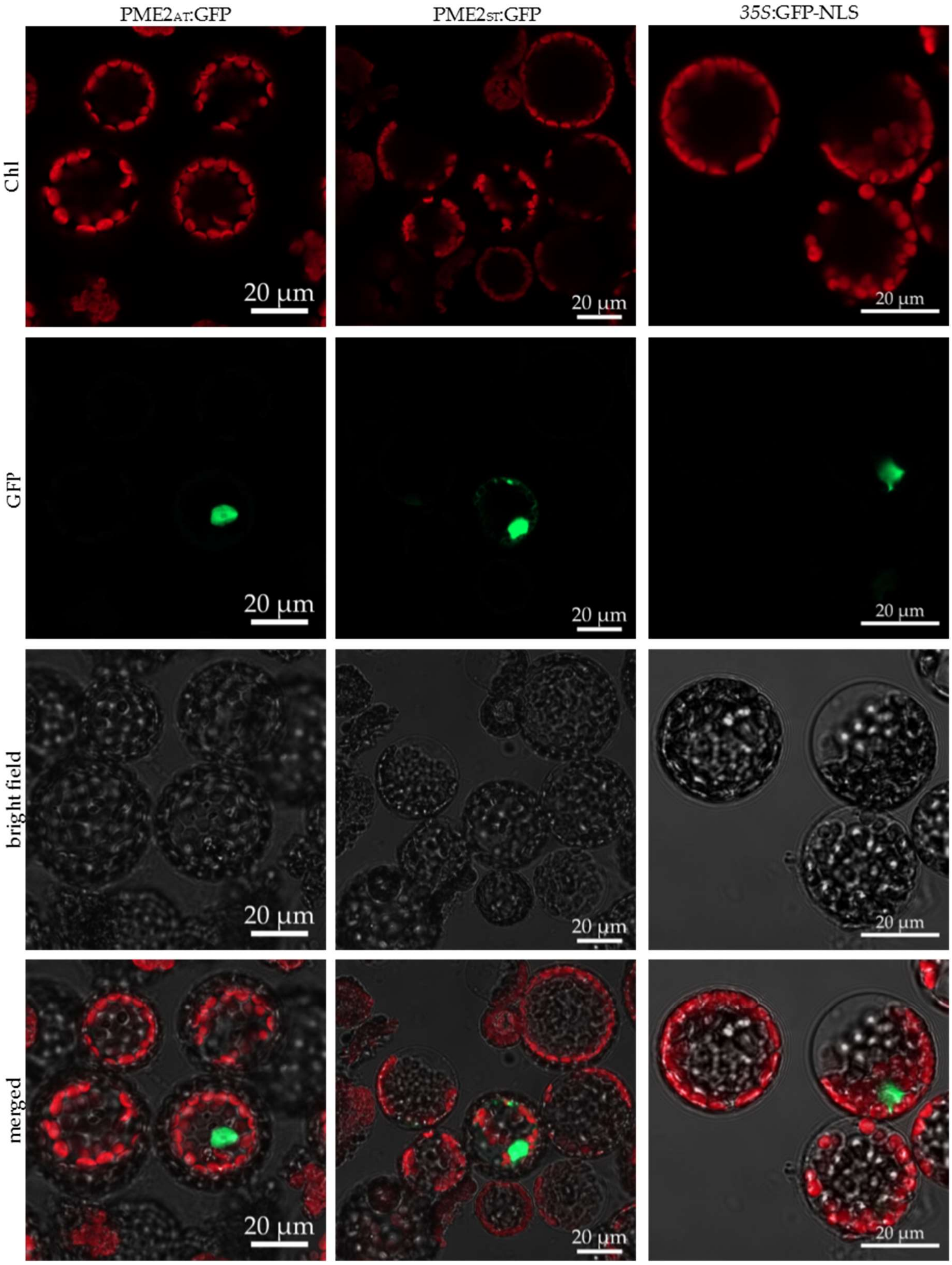

Figure S2. PME2ST and PME2AT are translocated to the nucleus of mesophyll protoplasts. Mesophyll protoplasts of *Nicotiana occidentalis* were transformed with the plasmid pGGZ001 encoding C-terminal GFP-tagged PME2ST (left column), PME2AT (middle column) or GFP-NLS (right column) as a control for nuclear localization. Expression of the transgenes was under the control of a *35S* promoter. The upper panel shows autofluorescence of chloroplasts (Chl), the second panel the signal derived from GFP, the third panel the bright field image and the last panel an overlay of all images (merged). Protoplasts transformed with the PME2ST expressing vector showed frequently shrinkage and cell disruption (arrows in the middle column). Microscopic analysis was performed with a Zeiss LSM 800.

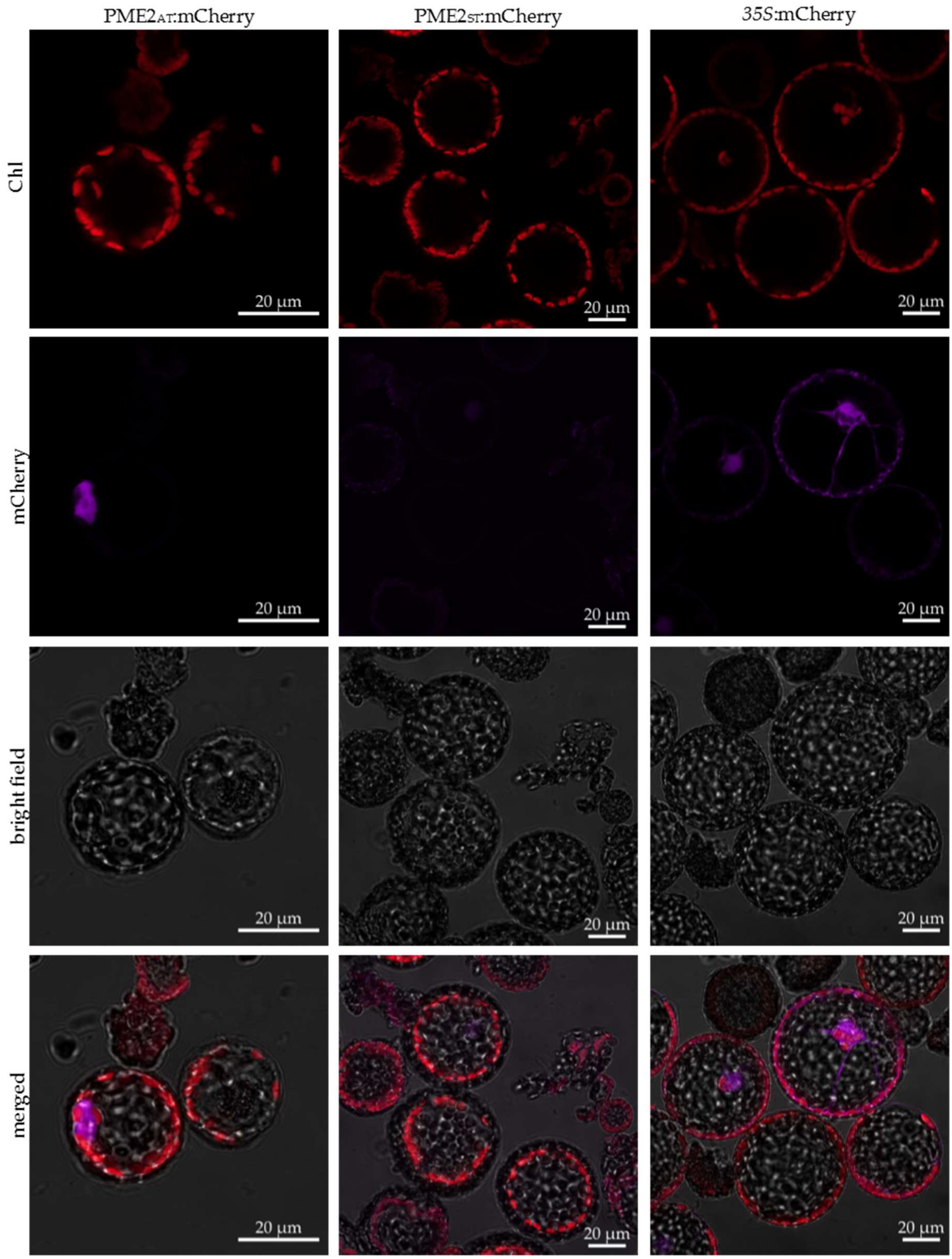

Figure S3. PME2ST and PME2AT are translocated to the nucleus of mesophyll protoplasts. Mesophyll protoplasts of *Nicotiana occidentalis* were transformed with the plasmid pGGZ001 encoding C-terminal mCherry-tagged PME2ST (left column), PME2AT (middle column) or mCherry (right column) as a control. Overexpression of the transgenes was under the control of a *35S* promoter. The upper panel shows autofluorescence of chloroplasts (Chl), the second panel the signal derived from mCherry, the third panel the bright field image and the last panel an overlay of all images (merged). Protoplasts transformed with the PME2ST expressing vector showed frequently shrinkage and cell disruption (arrows in the middle column). Microscopic analysis was performed with a Zeiss LSM 800.

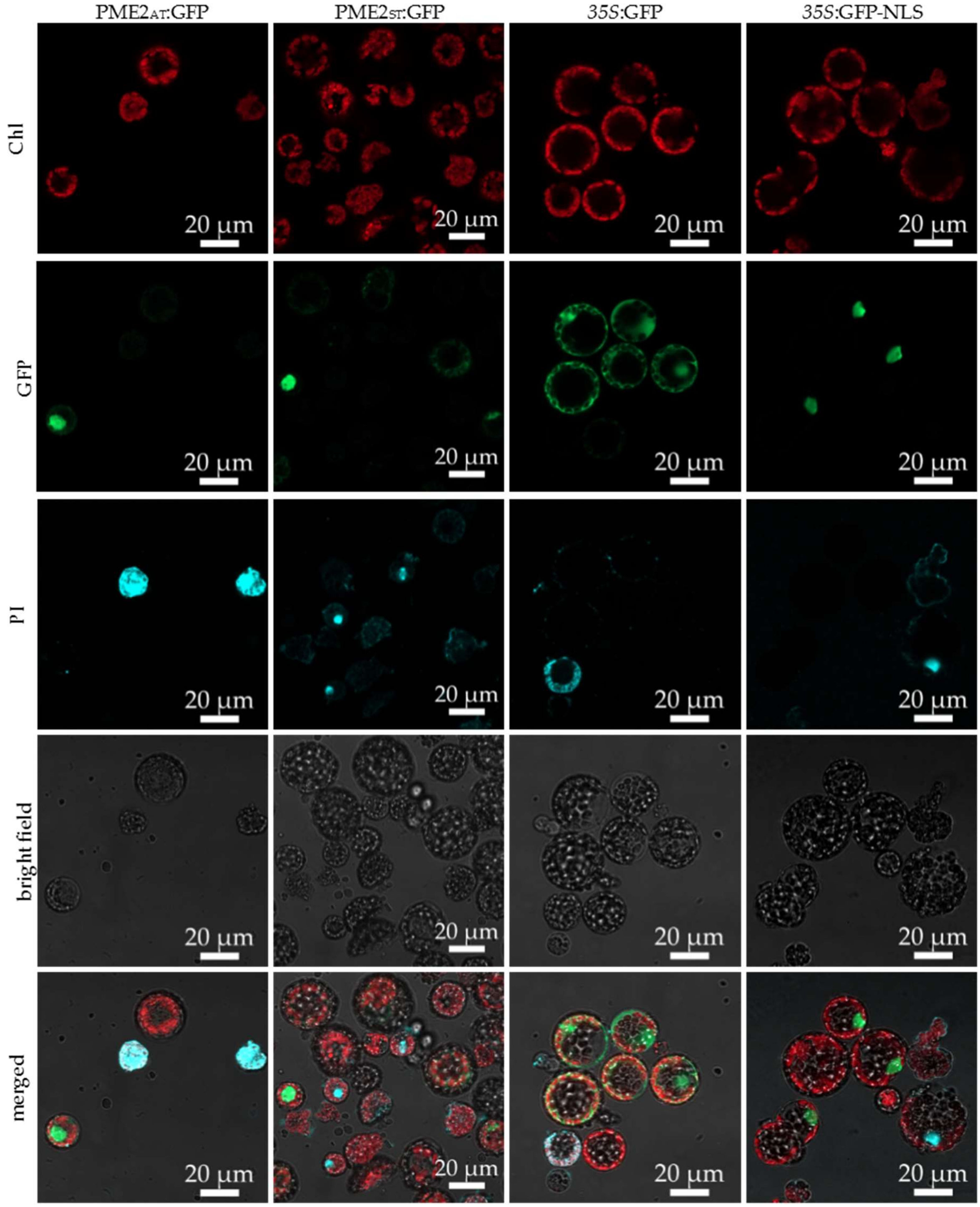

Figure S4. Mesophyll protoplasts of *Nicotiana benthamiana* were transformed with the plasmid pGGZ001 encoding C-terminal GFP-tagged PME2$_{ST}$ (left row), PME2$_{AT}$ (second row), only GFP (third row) or GFP-NLS (fourth row) under the control of a *35S* promoter and stained with propidium iodide (PI) as a cell viability marker. Death cells show a propidium iodide signal but no PME2 expression. Microscopic analysis was performed with a Zeiss LSM 800.

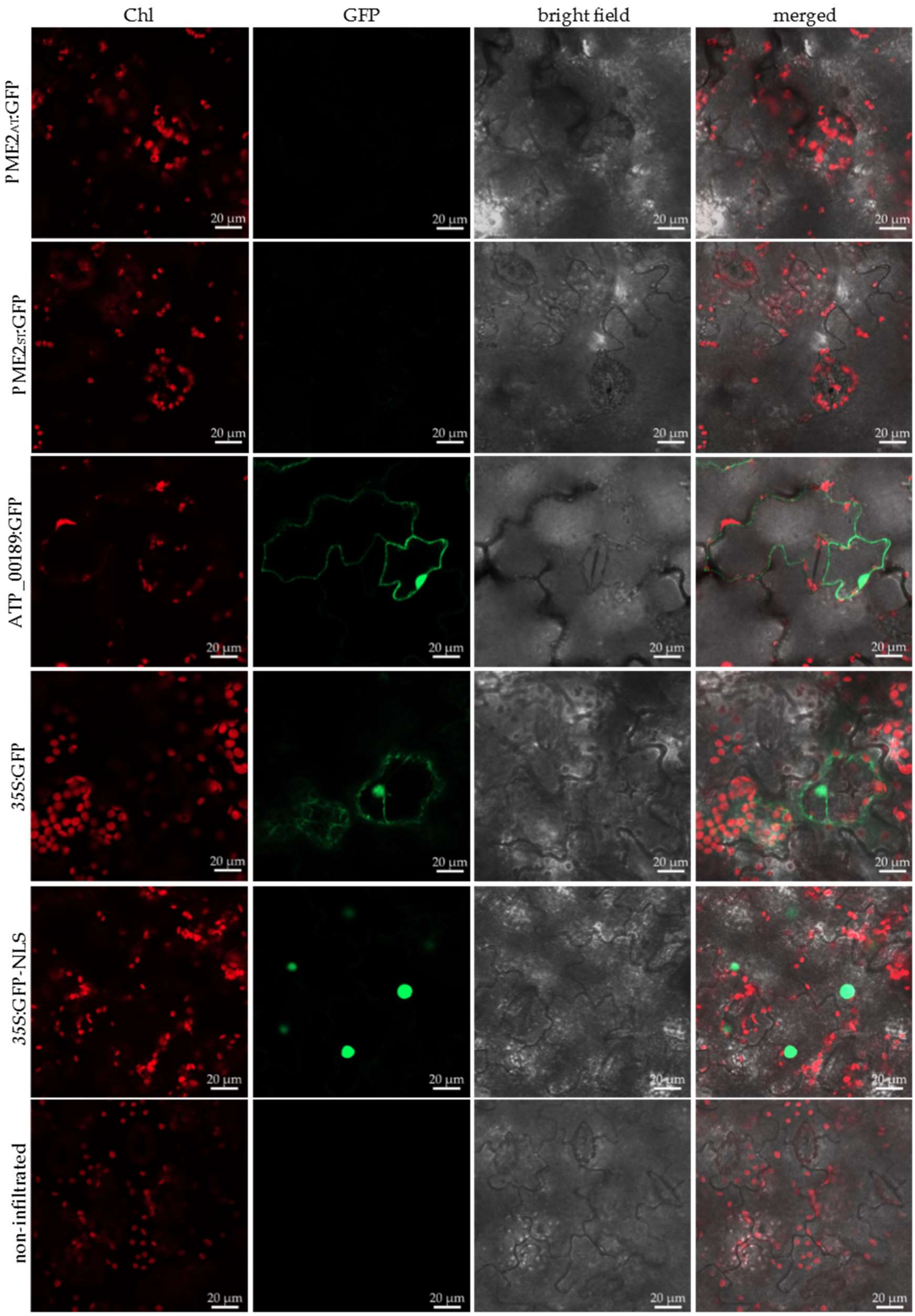

Figure S5. Abaxial leaf side of *Nicotiana benthamiana* leaves agroinfiltrated with the plasmid pGGZ001 encoding C-terminal GFP-tagged PME2$_{AT}$, PME2$_{ST}$, ATP_00189, only GFP or GFP-NLS under the control of a *35S* promoter. While transiently expressed PME2$_{AT}$ and PME2$_{ST}$ are not detectable, the SAP11-like effector protein ATP_00189 as well as the GFP controls are detectable in leaf epidermal cells. A non-infiltrated abaxial leaf side of *N. benthamiana* is shown as control. Imaging was performed with a Zeiss LSM 800.
